# Mesenteric IL-10-producing CD5^+^ regulatory B cells suppress cow’s milk casein-induced allergic responses in mice

**DOI:** 10.1038/srep19685

**Published:** 2016-01-20

**Authors:** A-Ram Kim, Hyuk Soon Kim, Do Kyun Kim, Seung Taek Nam, Hyun Woo Kim, Young Hwan Park, Dajeong Lee, Min Bum Lee, Jun Ho Lee, Bokyung Kim, Michael A. Beaven, Hyung Sik Kim, Young Mi Kim, Wahn Soo Choi

**Affiliations:** 1 School of Medicine, Konkuk University, Chungju 380-701, Korea; 2 Laboratory of Molecular Immunology, National Heart, Lung, and Blood Institute, National Institutes of Health, Bethesda, MD20892; 3 School of Pharmacy, Sungkyunkwan University, Suwon 440-746, Korea; 4 College of Pharmacy, Duksung Women’s University, Seoul 132-714, Korea

## Abstract

Food allergy is a hypersensitive immune reaction to food proteins. We have previously demonstrated the presence of IL-10-producing CD5^+^ B cells and suggested their potential role in regulating cow’s milk casein allergy in humans and IgE-mediated anaphylaxis in mice. In this study, we determined whether IL-10-producing CD5^+^ regulatory B cells control casein-induced food allergic responses in mice and, if so, the underlying mechanisms. The induction of oral tolerance (OT) by casein suppressed casein-induced allergic responses including the decrease of body temperature, symptom score, diarrhea, recruitment of mast cells and eosinophils into jejunum, and other biological parameters in mice. Notably, the population of IL-10-producing CD5^+^ B cells was increased in mesenteric lymph node (MLN), but not in spleen or peritoneal cavity (PeC) in OT mice. The adoptive transfer of CD5^+^ B cells from MLN, but not those from spleen and PeC, suppressed the casein-induced allergic responses in an allergen-specific and IL-10-dependent manner. The inhibitory effect of IL-10-producing CD5^+^ B cells on casein-induced allergic response was dependent on Foxp3^+^ regulatory T cells. Taken together, mesenteric IL-10-producing regulatory B cells control food allergy via Foxp3^+^ regulatory T cells and could potentially act as a therapeutic regulator for food allergy.

The prevalence of food allergy, an adverse immune reaction to allergenic food proteins^1^,^2^, is increasing and now affects approximately 6–8% of children in the United States of America. Peanut, milk, egg, and shellfish are well recognized as allergens that are responsible for allergic symptoms in patients with diseases such as gastrointestinal food allergy, atopic dermatitis, and anaphylaxis^3^,^4^. Among them, cow’s milk allergy (CMA) accounts for 2.5–5% of all allergic diseases and is the one most commonly associated with anaphylaxis and fatalities^5^,^6^,^7^,^8^. Cow’s milk protein consists of approximately 80% casein and 20% whey proteins and the major allergenic proteins have been identified within these two groups of proteins^9^,^10^.

The food allergic reactions have been classified under three types, “IgE-mediated” (type I reaction), “non-IgE-mediated (i.e. cell-mediated)”, and “combined IgE- and cell-mediated” types^11^. While the most common form of food allergy is IgE-mediated^12^, other immunoglobulins (Ig) such as IgG1 have been implicated in non-IgE-mediated and the mixed IgE/cell-mediated forms of food allergy^13^. Gastrointestinal food allergy belongs to the mixed type and the majority of children with CMA have gastrointestinal symptoms^14^.

The various therapies proposed include use of antihistamines, corticosteroids, antagonists against leukotrienes, and humanized anti-IgE antibody^15^. These therapies however are palliative rather than curative. Allergen-specific immunotherapy (AIT), also called hyposensitization, with incremental increases in dose of allergen was designed to induce specific allergy tolerance in patients with the aim of curing allergic disease instead of alleviating symptoms. Recent publications suggest that AIT is associated with recruitment of Foxp3^+^ regulatory T cells and IL-10-producing B cells, suppression of IgE, induction of IgG4, and suppression of eosinophil and mast cell activity in allergic tissues^16^. However, the mechanisms underlying these AIT related events have not been clarified.

Active B cells (B2 cells) positively regulate adaptive immune responses by producing antibody (Ab) and act as antigen-presenting cells to help induce optimal antigen-specific CD4^+^ T-cell activation^17^,^18^,^19^. However, over the past 30 years, the negative role of unique B cell subsets has also been recognized in mouse autoimmunity and allergic-inflammation models^20^,^21^. Further studies indicate that a specific B subsets including CD5^+^, CD1d^hi^CD5^+^, and T2-MZP inhibit immune responses through the production of IL-10, and thus named regulatory B (Breg) cells or B10 cells^22^,^23^. These cells are reported to suppress mouse autoimmunity and allergic inflammation in disease models that include contact hypersensitivity (CHS), experimental autoimmune encephalomyelitis (EAE) and systemic lupus erythematosus (SLE)^18^,^24^,^25^. We previously described the potential inhibitory role of IL-10-producing CD5^+^ B cells in human food allergic patients^26^,^27^ and in IgE-mediated allergic responses^28^. However, it is still unclear whether or not IL-10-producing CD5^+^ B cells suppress food allergic responses and, if so, by what mechanism.

In this study, we report that MLN (mesenteric lymph node)-derived IL-10-producing CD5^+^ B cells can suppress casein-induced allergy in a mouse model. The results demonstrate for the first time that this subset of CD5^+^ B suppresses casein-induced allergic responses via induction of Foxp3^+^ regulatory T cells in an IL-10-dependent manner.

## Results

### The population of IL-10^+^CD5^+^ B cells is increased in casein-induced oral tolerant mice

**Figure 1:**
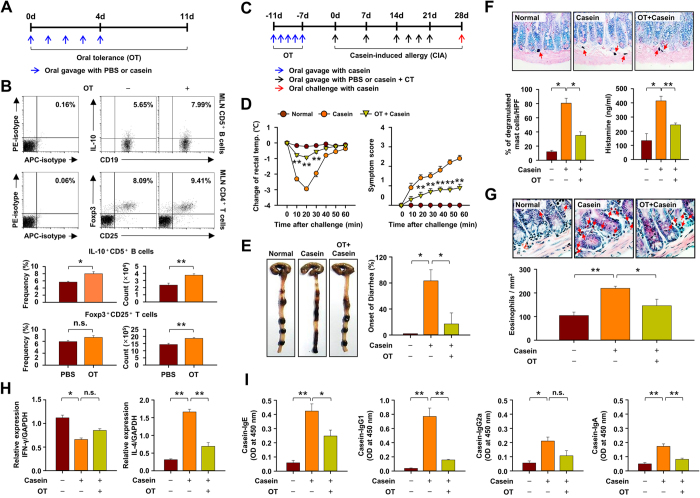
The population of IL-10-producing CD5^+^ B cell is increased in casein-induced oral tolerant mice. **(A)** Oral tolerance (OT) was induced with five administrations of 1 mg casein for 5 days before the induction of casein allergy. **(B)** Number of IL-10-producing CD5^+^ B cells and Foxp3^+^ regulatory T cells in MLN tissues after antigen pre-treatment. Data are mean ± SEM (n = 8). **(C–E)** Food allergy was estimated by changes in body temperature and systemic symptom score, occurrence of diarrhea. Data show the mean ± SEM from three independent experiments (n = 5 per group for each experiment). The statistical differences between the Casein and OT+Casein group are shown **(D)**. **(F)** Percentage of degranulated mast cells in jejunum (toluidine blue-stained sections) and serum histamine levels. **(G)** Number of eosinophils in jejunum (hematoxylin and eosin-stained sections). **(H)** Expressions of IFN-γ and IL-4 mRNA in jejunum using RT-PCR. **(I)** Measurements of casein-specific IgE, IgG1, IgG2a and IgA antibodies in serum by ELISA. **(F–I)** Results represent mean ± SEM (n = 8). **p < 0.01; *p < 0.05; n.s., not significant.

Regulatory T (Treg) cells are reported to participate in the induction of oral tolerance (OT) in a murine model^6^,^29^,^30^, but whether regulatory B (Breg) cells play an additional complementary role is unknown. We have investigated this possibility in a casein-induced allergy (CIA) model in mice. The population and frequency of IL-10-producing CD5^+^ B cells and Foxp3^+^ Treg cells increased in MLN of OT mice (as per Fig. 1A) when compared to the PBS-treated normal mice (Fig. 1B), but no significant difference was observed in spleen or other tissues (data not shown). Comparison of various allergic symptoms during CIA (Fig. 1C) revealed that the decline in rectal temperature, allergic symptom score, and onset of diarrhea were substantially suppressed in the OT mice compared to non-tolerant mice (Fig. 1D,E). Other relevant events including degranulation of mast cells (Fig. 1F) and infiltration of eosinophils (Fig. 1G) into jejunum after induction of CIA were significantly inhibited in OT mice as well. Furthermore, expression of a representative Th2-related cytokine, IL-4 mRNA, was largely suppressed in the jejunum of OT mice whereas expression of the Th1-related cytokine, IFN-γ, was minimally effected (Fig. 1H). Serum histamine and casein-specific antibodies (IgE, IgG1, IgG2a and IgA) were also much reduced in OT mice (Fig. 1F,I). These data suggest the possible involvement of IL-10^+^CD5^+^ B cells in addition to Foxp3^+^ Treg cells in the induction of OT and the suppression of food allergy in mice.

### The population of IL-10^+^CD5^+^ B cells is increased in mesenteric lymph node, but not in spleen and peritoneal cavity, by casein challenge

**Figure 2:**
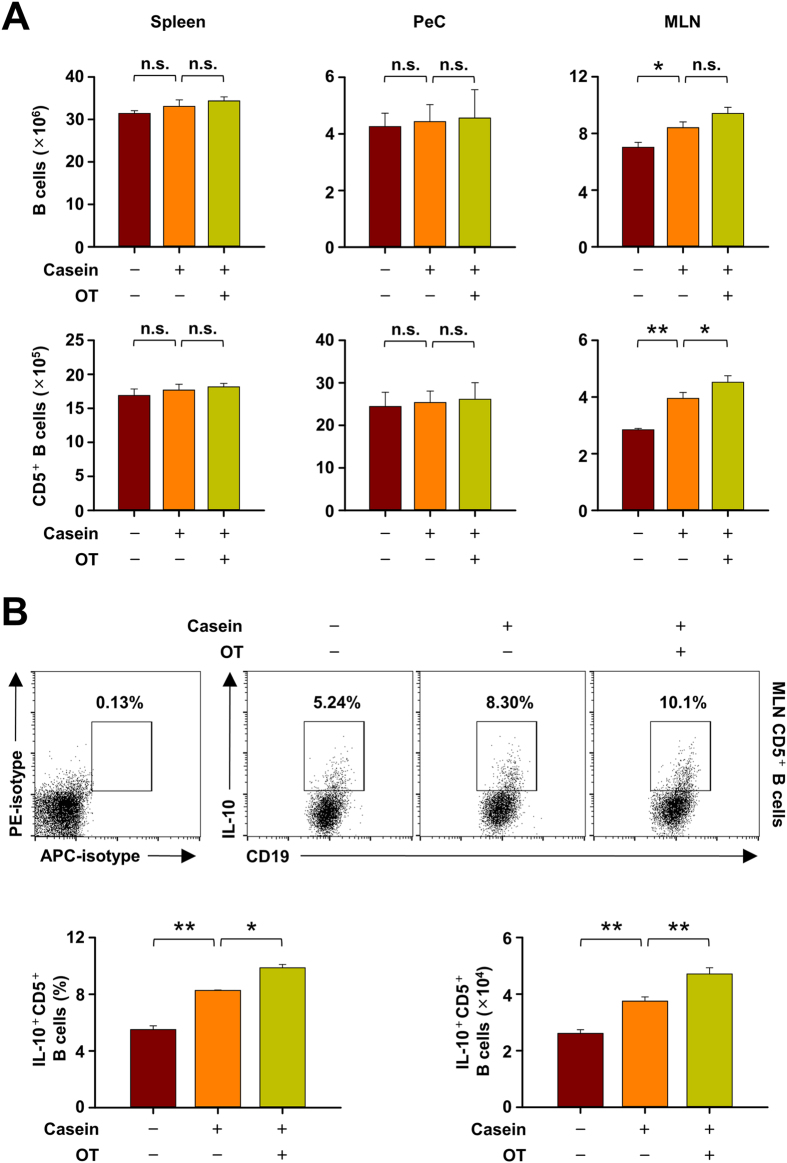
The increase of IL-10-producing CD5^+^ B cell is observed in mesenteric lymph node (MLN), but not in spleen and peritoneal cavity (PeC). (**A**) Spleen, PeC and MLN cells were isolated, determined the number of whole B cells and CD5^+^ B cells in normal, casein-induced allergy (CIA), and oral tolerant (OT) mice by flow cytometry. (**B**) MLN cells stimulated in vitro 5 h with LPIB solution (LPS + PMA + ionomycin + Brefeldin A) and stained for intracellular IL-10. The percentage and number of IL-10^+^ cells among CD5^+^ B cells in MLNs were counted by flow cytometry. Data are mean ± SEM (n = 8). Statistical comparisons were made with ANOVA followed by Duncan’s test. **p < 0.01; *p < 0.05; n.s., not significant.

On investigating the changes in population of all B cells and CD5^+^ B cells specifically in various locations, we observed that the numbers of both populations were increased by CIA in MLN, but not in spleen and peritoneal cavity (PeC) (Fig. 2A). In addition, the population and percentage of IL-10-producing CD5^+^ B cells were significantly elevated by CIA and more so in OT mice (Fig. 2B), suggesting again that IL-10-producing CD5^+^ B cells in MLN are possibly associated with the regulation of food allergy in mice.

### The adoptive transfer of CD5^+^ B cells from MLN, but not from spleen and peritoneal cavity, inhibits casein-induced allergic responses

**Figure 3:**
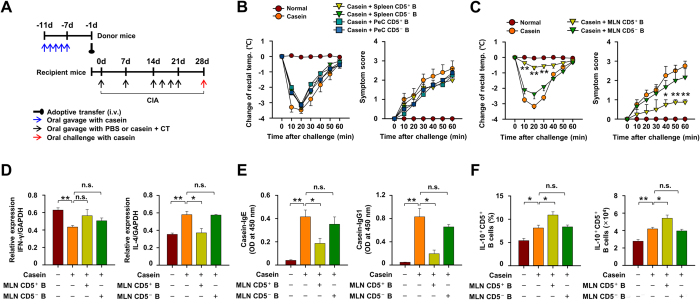
The adoptive transfer of CD5^+^ B cells from mesenteric lymph node (MLN), but not from spleen and peritoneal cavity (PeC), inhibit casein-induced allergic responses. (**A**) CD5^+^ or CD5^−^ B cells of MLN, spleen and PeC from casein-sensitized donor mice were transferred to recipient mice one day before induction of casein-induced allergy (CIA). Rectal temperatures and systemic symptom scores following CIA in WT mice and where indicated adoptive transfer of (**B**) spleen/PeC CD5^+^ or CD5^−^ B cells and (**C**) MLN CD5^+^ or CD5^−^ B cells. (**D**) Expression of IFN-γ and IL-4 mRNA levels in jejunum using RT-PCR. (**E**) Measure of casein-specific IgE and IgG1 antibodies in serum by ELISA. (**F**) The percentage and number of IL-10^+^ cells among CD5^+^ B cells in MLNs were counted. Data are mean ± SEM (n = 8). The statistical differences between the Casein and other groups are shown (**B–C**). **p < 0.01; *p < 0.05; n.s., not significant.

CD5^+^ and CD5^−^ B cells derived from donor mice with OT to casein were transferred into naïve recipient mice which were then subjected to CIA (Fig. 3A). The adoptive transfer of CD5^+^ B cells from MLN, but not those from PeC and spleen, of OT mice suppressed the decrease of rectal temperature, symptom scores (Fig. 3B,C), the expression of Th2 cytokine IL-4 in jejunum and partially reversed the reduction of Th1 cytokine IFN-γ, but not to a significant extent (Fig. 3D), following induction of CIA in the recipient mice. The adoptive transfer of MLN CD5^+^ B cells also reduced the CIA-induced increase in levels of serum IgE and IgG1 (Fig. 3E) and resulted in significant elevation in the number and frequency of IL-10-producing CD5^+^ B cells in MLN (Fig. 3F). Of note, the adoptive transfer of CD5^-^ B cells had no effect in any of the above experiments (Fig. 3B–F). These results demonstrated that MLN CD5^+^, but not CD5^−^, B cells regulate casein-induced food allergic responses in mice.

### The suppressive effect of CD5^+^ B cells on CIA responses is casein-specific

**Figure 4:**
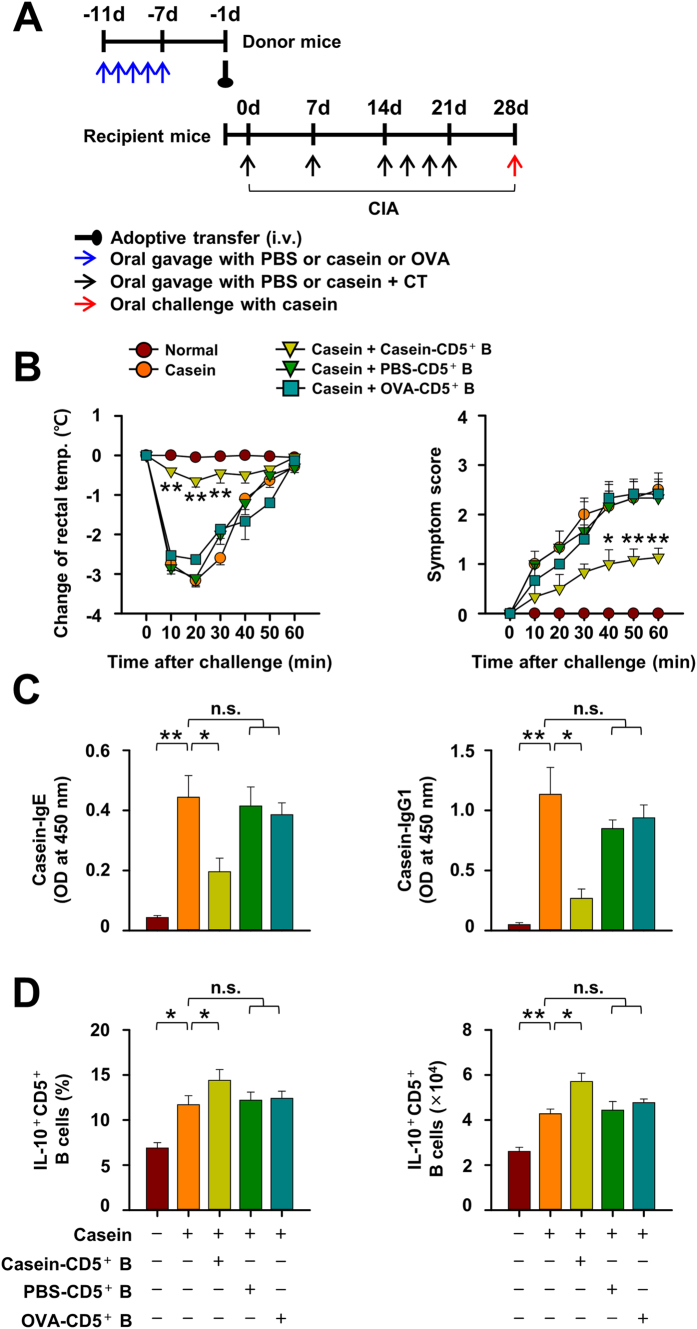
The suppressive effect of CD5^+^ B cells on casein-induced allergic (CIA) responses is allergen-specific. **(A)** Mesenteric lymph node (MLN) CD5^+^ B cells from normal, casein, or ovalbumin (OVA)-sensitized donor mice were transferred to recipient mice before induction of CIA. **(B)** The severity of CIA was evaluated by changes in body temperatures and symptom scores in the recipient mice. **(C)** Casein-specific IgE and IgG1 antibodies were measured by ELISA. **(D)** The percentage and number of IL-10-producing CD5^+^ B cells in MLNs were counted by flow cytometry. Data are mean ± SEM (n = 8). The statistical differences between the Casein and other groups are shown (B). **p < 0.01; *p < 0.05; n.s., not significant.

We next investigated whether the inhibition of casein-induced allergic responses by the adoptive transfer of MLN CD5^+^ B cells is casein-specific or not. Inhibition was observed only on adoptive transfer of MLN CD5^+^ B cells from casein-sensitized donor mice, but not by transfer of MLN CD5^+^ B cells from PBS-treated or OVA-sensitized donor mice (Fig. 4A,B). This was true for the decrease in rectal temperature and systemic symptom scores (Fig. 4B), serum levels of casein-specific IgE and IgG1 levels (Fig. 4C), and the increase in the population and number of MLN IL-10-producing CD5^+^ B cells in CIA mice (Fig. 4D). The adoptive transfer of MLN CD5^+^ B cells from PBS-treated or OVA-sensitized mice had no significant effect as compared to the adoptive transfer of MLN CD5^+^ B cells from casein-sensitized mice. The results imply that inhibition of CIA occurs only on adoptive transfer of casein-sensitized MLN CD5^+^ B cells in an allergen-specific manner.

### IL-10 from MLN CD5^+^ B cells is critical for the suppression of casein-induced allergic responses

**Figure 5:**
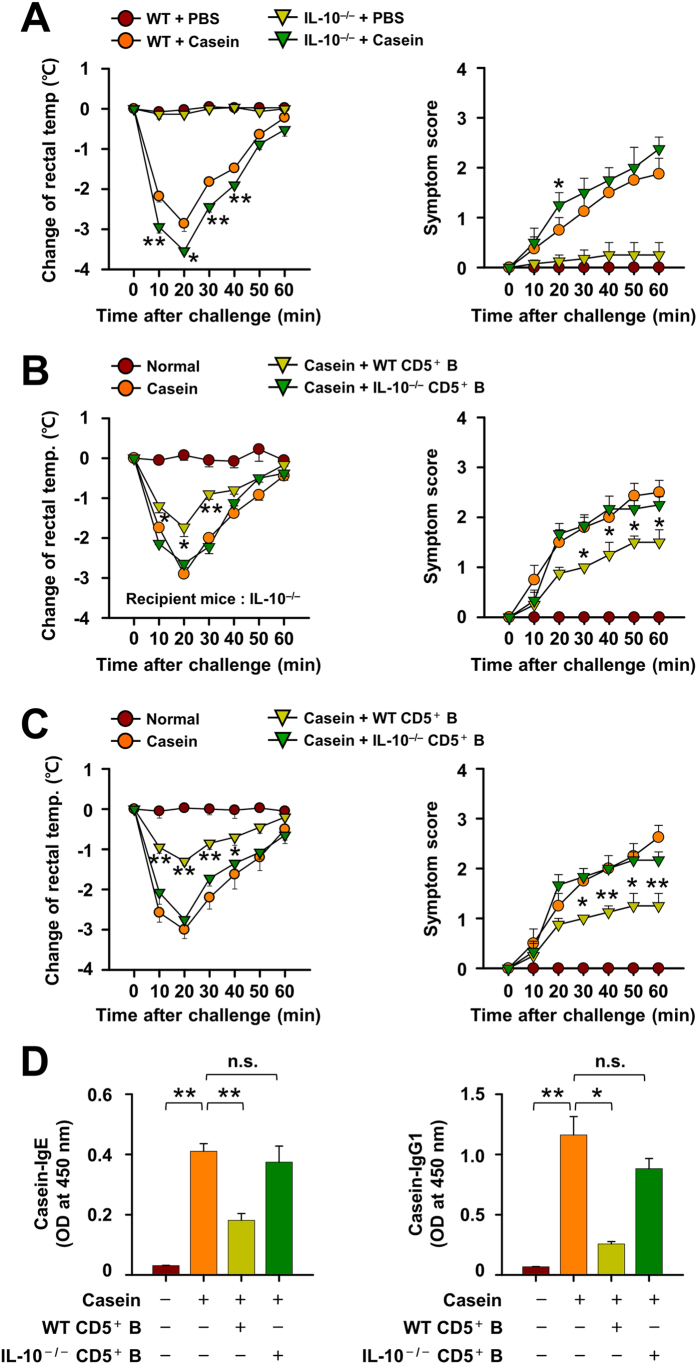
IL-10 from mesenteric lymph node (MLN) CD5^+^ B cells is critical for the suppression of casein-induced allergy (CIA). **(A)** The severity of CIA was estimated by changes in body temperatures and systemic symptom scores in the WT or IL-10-deficient mice. **(B,C)** MLN CD5^+^ B cells from WT or IL-10^−/−^donor mice were transferred to recipient IL-10-deficient mice before the induction of food allergy. The severity of CIA was estimated by changes in body temperatures and systemic symptom scores. **(D)** Casein-specific IgE and IgG1 antibodies were measured by ELISA. Data are mean ± SEM (n = 8). The statistical differences between the WT+Casein and IL-10^-/-^+Casein groups **(A)** or the Casein and other groups are shown **(B,C)**. **p < 0.01; *p < 0.05; n.s., not significant.

The importance of IL-10-producing CD5^+^ B cells in attenuating CIA was evaluated using IL-10 deficient (IL-10^−/−^) mice. The decrease in rectal temperatures and systemic symptom scores during CIA were more pronounced in IL-10^−/−^ mice than in WT mice (Fig. 5A). Of note, while the adoptive transfer of MLN CD5^+^ B cells from casein-sensitized WT mice to IL-10^−/−^ recipient mice inhibited CIA reactions, those from IL-10^−/−^ mice did not (Fig. 5B). The apparent requirement for IL-10 producing CD5^+^ B cells was further confirmed in WT mice by the adoptive transfer of WT or IL-10^−/−^ MLN CD5^+^ B cells where the latter cells failed to prevent the drop in rectal temperature (Fig. 5C) and the increase of casein-specific serum IgE and IgG1 on induction of CIA (Fig. 5D). Additionally, we confirmed that IL-10-producing CD5^+^ B cells were minimal in CD19^−/−^mice which, as previously reported^31^, are incapable of generating this subset of B cells (see supplementary Fig. S1A online). The CIA-induced decrease in rectal temperatures and systemic symptom scores were even more severe in CD19^−/−^CIA mice than WT CIA mice (see supplementary Fig. S1B online). Collectively, the results strongly suggest that IL-10-producing subset of CD5^+^ B cells were responsible for the suppressive effect of MLN CD5^+^ B cells on CIA responses.

### IL-10-producing CD5^+^ B cells suppresses CIA through the induction of FoxP3^+^ regulatory T cells

**Figure 6:**
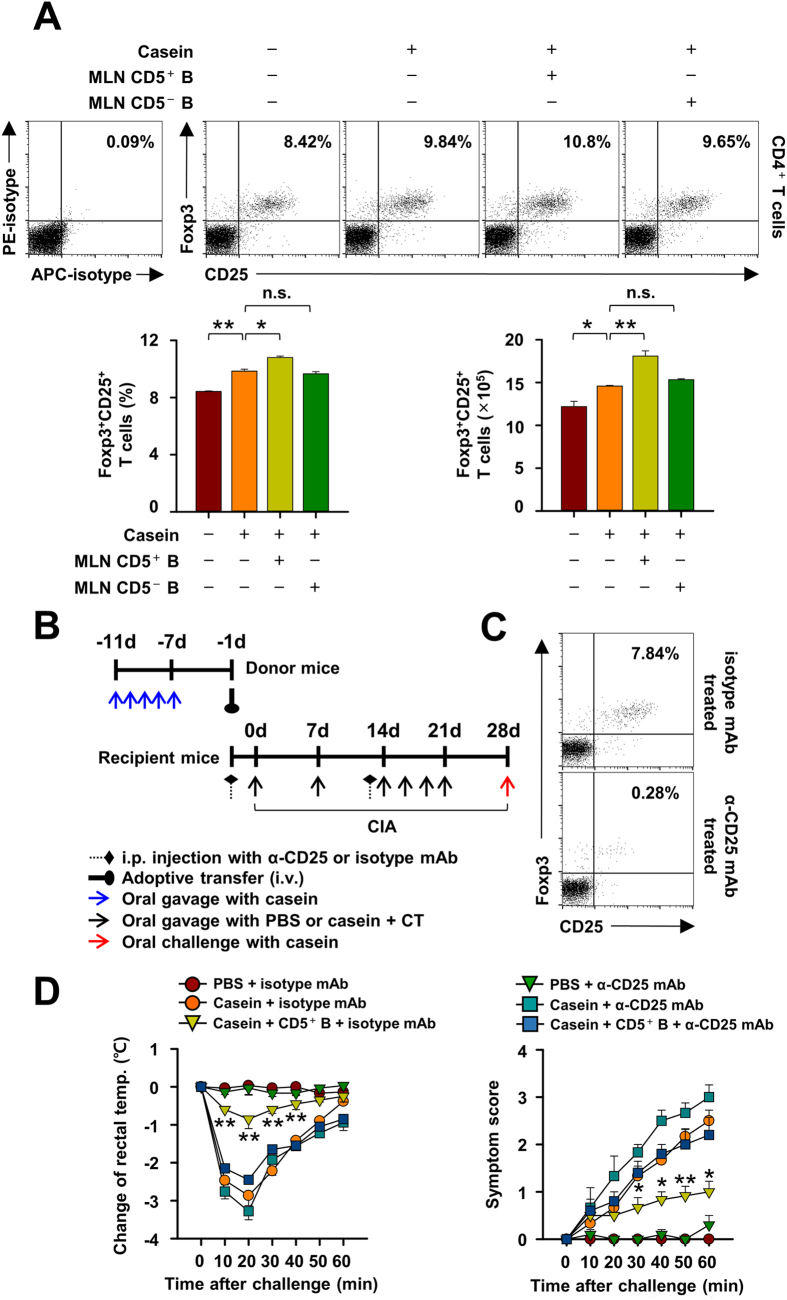
Foxp3^+^ CD25^+^ regulatory T cells is essential for the suppression of casein-induced allergy (CIA) by IL-10-producing CD5^+^ B cells in mice. (**A**) Mesenteric lymph node (MLN) CD5^+^ or CD5^−^ B cells from donor mice were transferred to recipient mice before the induction of casein allergy. The populations of Foxp3^+^ CD25^+^ T cells in MLNs were measured. (**B,C**) CD25^+^ T cells were depleted by the intraperitoneal injection of anti-CD25 mAb (PC61) one and thirteen days before the induction of CIA. The representative images are shown for the population of Foxp3^+^ CD25^+^ T cells in MLN. (**D**) MLN CD5^+^ B cells from casein-sensitized donor mice were transferred to WT or CD25^+^ T cell-depleted mice before the induction of CIA. The severity of CIA was estimated by changes in rectal temperatures and clinical symptoms. Data are mean ± SEM (n = 8). The statistical differences between the Casein+isotype mAb and other groups are shown (**D**). **p < 0.01; *p < 0.05; n.s., not significant.

IL-10-producing regulatory B cells are reported to suppress allergen-induced airway inflammation via the induction of Foxp3^+^ Treg cells in lung^31^. We have investigated whether Foxp3^+^ Treg cells have a similar role in our model and found that the prevalence and number of Foxp3^+^ Treg cells in the MLN of mice with CIA increased after adoptive transfer of casein-sensitized MLN CD5^+^ B cells, but not MLN CD5^−^ B cells, as compared to control mice without adoptive transfer (Fig. 6A). However, no meaningful change was observed in Foxp3^+^ Treg cell population after adoptive transfer of CD5^+^ B cells from the spleen (data not shown), suggesting that a MLN-specific induction of Foxp3^+^ Treg cells after the adoptive transfer of CD5^+^ B cells could be linked to the inhibitory effect of MLN IL-10-producing CD5^+^ B cells.

To verify the relevance of Foxp3^+^ Treg cells to the inhibition of CIA, we depleted Foxp3^+^ CD25^+^ Treg cells by using anti-CD25 mAb (clone PC61) in recipient mice (Fig. 6B). The accumulation of MLN CD4^+^CD25^+^Foxp3^+^ regulatory T cells during CIA was largely prevented by this treatment (Fig. 6C). The systemic symptoms of CIA and decline in rectal temperature were slightly exacerbated, but not significantly, by anti-CD25 mAb treatment as compared to untreated mice (Fig. 6D). However, the suppressive effect of the MLN CD5^+^ B cells on CIA was not apparent in the Foxp3^+^ Treg cell-depleted mice (Fig. 6D), suggesting that Foxp3^+^ Treg cells are essential for the suppression of CIA by IL-10-producing MLN CD5^+^ B cells.

## Discussion

The number of hospitalizations for food-related anaphylaxis has increased rapidly over the past decade^32^,^33^ with the respiratory and circulatory systems being primarily affected^34^. Common allergens include milk, egg, peanut, and shellfish proteins^35^. Among these cow’s milk is a frequent cause of allergy in infants and young children^36^ with eczema and itching being the most common symptoms^4^,^5^,^36^,^37^. However, in all groups of patients with milk allergy, casein is the principal allergen in cow’s milk^36^,^38^.

Therapeutic approaches for food allergy can be classified into allergen-specific immunotherapy and nonspecific therapies. Allergen-specific immunotherapy includes oral, sublingual, and epicutaneous immunotherapy to expose patients to increasing doses of allergen. Nonspecific therapy might include anti-histamine drugs, anti-IgE antibodies, Toll-like receptor agonists, and probiotics^33^. Despite the advantages of allergen-specific immunotherapy, some patients show no improvement or it proves to be inadvisable due to adverse reactions^39^.

Medawar and Burnet define immune tolerance as “a state of indifference or non-reactivity towards a substance that would normally be expected to excite an immunological response”^40^. Induction of OT is a well-recognized therapeutic procedure to establish peripheral immune tolerance^41^. Treg cells appear to perform a key role in OT in animal models^6^,^29^,^30^,^42^. During allergen-specific immunotherapy in humans, Treg cells are generated and suppress Th2 cells and other effector cells in an allergen-specific manner^39^. In mice, depletion of Treg cells before sensitization to food protein augments the elevation of allergen-specific IgE levels and mast cell degranulation to subsequent oral challenge with food protein^43^. The adoptive transfer of Treg cells isolated from tolerized mice prevented allergic responses in naïve mice^44^,^45^. Other evidence suggests that Treg cells contribute to oral tolerance in the gastrointestinal tract^45^,^46^ and that Treg cells from MLN are largely involved in the induction of oral tolerance^7^,^47^.

The involvement of IL-10-producing B cell subsets in food allergies is unclear although IL-10–producing Breg cells enable suppression of anaphylaxis, EAE and collagen-induced arthritis in animal models^24^,^28^,^48^. Also, we have suggested that IL-10-producing CD5^+^ B cells could play a critical role in the suppression of allergic responses in patients allergic to cow’s milk^26^,^27^. In this study, we demonstrate that IL-10-producing CD5^+^ B cell may promote the development of oral tolerance to casein from cow’s milk and describe some of the underlying mechanisms involved. We find that the population of IL-10-producing CD5^+^ B cells is increased in MLN, but not in spleen and PeC, of casein-tolerized mice (Fig. 2). This is consistent with previous reports that oral tolerance cannot be induced in mice lacking MLN^49^,^50^ which appears to be the critical lymphoid tissue. Furthermore, the adoptive transfer of mesenteric CD5^+^ B cells, but not CD5^−^ B cells nor CD5^+^ B cells from spleen or PeC, from casein-tolerized mice suppressed CIA symptoms as well as the induction of casein-specific antibodies and IL-4 in naïve mice (Fig. 3C–E). As CD5^+^ B cells, but not CD5^−^ B cells, produce IL-10^28^ and CD5^−^ or IL-10-deficient CD5^+^ B cells do not suppress CIA (Fig. 5), IL-10 is a likely participant in the regulation of CIA in mice. Notably, the transfer of casein-sensitized MLN CD5^+^ B cells inhibited only casein-induced allergic responses (Fig. 4), indicating that the effect is allergen-specific.

**Figure 7:**
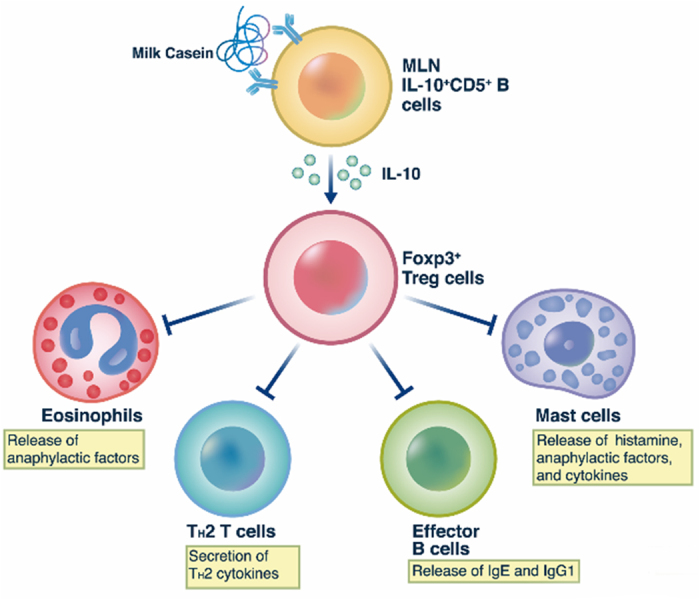
Proposed scheme for suppression of casein-induced allergy by IL-10-producing CD5^+^ B lymphocytes. MLN IL-10-producing CD5^+^ B cells induces Foxp3^+^ regulatory T (Treg) cells activation and then Treg cells suppress eosinophils, mast cells, T_H_2 responses, and effector B cells. The process is dependent on IL-10 produced by CD5^+^ B cells.

Our studies also provide evidence for the participation of Treg cells. Studies of other mouse models have demonstrated that B cell deficiency delays the appearance of Treg cells and IL-10 in the central nervous system during EAE. Cooperation between Breg and Treg cells is critical for suppression of EAE development and, as they act at different time points during EAE initiation and progression, these cells may have sequential functions^51^. Also, IL-10-producing Breg cells induce pulmonary infiltration of Treg cells which, in turn, inhibit allergic airway inflammation in mice^31^,^52^. Treg cells themselves inhibit the activation of effector cells by allergen^53^, suppress the influx of eosinophils and effector T cells into inflamed tissues^54^ and inhibit the activation of Th2 cells during allergic responses^46^. We find that Foxp3^+^ regulatory T cells are also essential for the suppression of CIA by IL-10-producing CD5^+^ B cells. The adoptive transfer of IL-10-producing CD5^+^ B cells, but not of CD5^−^ B cells, was found to increase the population of Foxp3^+^CD25^+^ Treg cells in MLN (Fig. 6A). Furthermore, the adoptive transfer of CD5^+^ B cells failed to suppress CIA in Foxp3^+^ CD25^+^ T cell-depleted mice (Fig. 6B–D) to further suggest that the suppressive actions of CD5^+^ B are dependent on Foxp3^+^ CD25^+^ T cells. While our studies demonstrate that Breg cell-derived IL-10 and Fox3^+^ Treg cells are required for development of OT, the exact manner of interaction between these two cell types is unknown but most likely involves the paracrine action of IL-10 on Foxp3^+^ CD25^+^ T cells within MLN (as depicted in Fig. 7).

Additional evidence that B cell-derived IL-10 inhibits CIA comes from studies with CD19-deficient (CD19^−/−^) mice. CD19 is an essential surface marker protein for modulating B cell responses^55^. Most B cell subsets are generated normally in CD19^−/−^mice^56^, but IL10-producing B cell subsets are barely detectable^25^,^57^ (see supplementary Fig. S1A online). CD19^−/−^mice have been used to examine the role of IL-10-producing Breg cells in various diseases^25^ such as contact hypersensitivity and EAE which are exacerbated in CD19^−/−^mice by the absence of IL-10-producing B cells^58^,^59^. The more pronounced allergic responses in CD19^−/−^CIA mice in our study (see supplementary Fig. S1B online) suggest also that IL-10-producing CD5^+^ B cells are critical for suppressing CIA responses in mice. However, a caveat in extrapolating observations from mouse models to the clinical situation is that the mechanisms of induction of food tolerance in naïve mice may differ from those in patients where tolerance is induced by reversal of an existing sensitivity to a food allergen. In addition, the underlying pathology or genetic predisposition may also affect the course of desensitization.

In conclusion, MLN-IL-10-producing CD5^+^ B cells suppressed CIA responses in an allergen-specific and Foxp3^+^ Treg cell-dependent manner. This interaction was associated with suppression of immune responses such as infiltration of eosinophils, release of Th2 cytokines, production of IgE and IgG1, mast cell degranulation, and secretion of histamine and anaphylactic factors (Fig. 7). Overall, our results suggest that IL-10-producing CD5^+^ B cells are associated with development of oral tolerance for foods and may provide new insight on therapeutic approaches for treatment of food allergies.

## Methods

### Mice

Wild-type (4-5-week-old female mice), IL-10^−/−^(*Il10*^*tm1Cgn*^) mice or CD19^−/−^(*Cd19*^*tm1(cre)Cgn*^) on C57BL/6 background and BALB/c mice were purchased from The Jackson Laboratory (Bar Harbor, ME). Mice were housed in a pathogen-free facility of Konkuk University (Seoul, Korea). All experiments were performed in accordance with the guidelines and protocols approved by the Institutional Animal Care and Use Committee (IACUC) at Konkuk University.

### Induction of casein-mediated food allergy in mice

Female mice were sensitized with 1 mg of casein (Sigma-Aldrich, St. Louis, MO) plus 10 μg of cholera toxin (CT, List Biological Laboratories, Inc., Campbell, CA) by oral administration (p.o.) in 200 μl PBS on days 0 and 7. After this, mice were orally boosted four times for another week, with 5 mg of casein plus 10 μg of CT. One week later, allergic responses were induced by 50 mg of casein in PBS p.o. To induce oral tolerance (OT), mice were administered 1 mg casein p.o. every day for 5 days.

Immediately after challenge, the severity of food allergy was assessed by the change in rectal temperature using a digital thermometer TESTO 925 (Testo AG, Germany). Symptom scores were as follows: 0, no symptoms; 1, scratching around nose and head; 2, puffiness around eyes and mouth; 3, wheezing, labored respiration, cyanosis around mouth and tail; 4, no activity after prodding, or tremor and convulsion; 5, death.

### Adoptive transfer of CD5^+^ B cells into mice

Donor mice received casein or ovalbumin at a dose of 1 mg/mouse or PBS daily for 5 days. Lymphocytes were isolated from spleen PeC and MLN from donor mice from which B cells were derived by presorting with CD19 microbeads (Miltenyi Biotec). CD5^+^ or CD5^−^ B cells were subsequently sorted by FACSAria (BD Biosciences, Franklin Lakes, NJ, USA). For adoptive transfer, the isolated CD5^+^ or CD5^−^ B cells (2 × 10^6^cells) were intravenously injected into recipient mice 1 day before the induction of casein-induced food allergy (CIA).

### *In vivo* CD25^+^ T cell depletion

Mice were intraperitoneally injected with 200 μg of anti-mouse CD25 mAb PC61 (eBioscience, San Diego, CA) at days -1 and 13 according to previous reports^7^,^60^. Cell depletion was confirmed by flow cytometry analysis.

### Flow cytometry analysis

Single-cell suspensions were isolated from spleen, PeC and MLN tissues. For detection of intracellular IL-10 in B cells, isolated cells were resuspended and incubated for 5h with LPS (10 μg/ml, Sigma), phorbol 12-myristate 13-acetate (PMA, 50 ng/ml, Sigma), ionomycin (500 ng/ml, Sigma), and brefeldin A (3 μg/ml, eBioscience). To detect intracellular Foxp3 in T cells, the isolated cells were resuspended and exposed to the same agents, except LPS, for 4 h. Before staining for cell surface makers, cells were blocked with anti-CD16/CD32 Fc-block (2.4G2, BD Biosciences). Cells were fixed and permeabilized with Cytofix/Cytoperm kit (BD Biosciences) and then were stained with anti-IL-10 (JES5-16E3, eBioscience) and Foxp3 (FJK-16s, eBioscience). Abs against mouse surface proteins were: CD4 (GK1.5), CD5 (53-7.3), CD19 (eBio1D3), CD25 (PC61.5), and B220 (RA3-6B2), all from eBioscience Inc. (San Diego, CA). Cells were analyzed with FACScalibur (Becton Dickinson, San Jose, CA) and FlowJo version 10 software (TreeStar).

### Measurement of casein-specific antibody in serum

Serum levels of casein-specific IgA, IgG1, IgG2a, and IgE were analyzed using ELISA. A microtiter plate was coated with 50 μg casein/well in buffer (0.1 M sodium carbonate, pH 9.5) and incubated for 18 h at 4 °C. After washing, plates were reincubated for 1h with a blocking PBS buffer, pH 7.0, containing 10% FBS. Serum samples were added to the wells and incubated for 2 h at room temperature (RT). Plates were washed three times with blocking buffer, incubated with 2 μg biotin-labeled rat anti-mouse IgE, IgG1, IgG2a or IgA (BD Biosciences, San Jose, CA) for 1 h at RT and then, after removal of supernatants, washed three times with the blocking buffer. Plates were further incubated for 30 min at RT after addition of a solution (100 μl/well) of streptavidin-HRP (BD Biosciences, San Jose, CA). This solution was replaced with a substrate solution (TMB; GenDEPOT, Barker, TX, USA) (100 μl/well). Absorbance was measured at 450 nm.

### Reverse transcriptase-polymerase chain reaction (RT-PCR)

Total RNA was isolated from jejunum by using Trizol reagent (Invitrogen, Carlsbad, CA, USA) and reverse transcribed by use of SuperScript first-strand synthesis system (Invitrogen, Carlsbad, CA). Primers used: mouse IFN-γ (forward, 5΄-AGGAAGCGGAAAAGGAGTCG-3΄; reverse, 5΄-GGGTCACTGCAGCTCTGAAT-3΄); IL-4 (forward, 5΄-TCACTGACGGCAC AGAGCTA-3΄; reverse, 5΄-TGCAGCTCCATGAG AACACT-3΄).

### Histological analysis of mast cells and eosinophils

After induction of CIA, isolated jejunums was fixed in 4% paraformaldehyde in PBS, dehydrated by incremental concentrations of ethanol (70 to 100%), washed twice with xylene for 3 min, and then embedded in paraffin. Paraffin sections, 5-μm thick, were stained with toluidine blue (for mast cells) or hematoxylin and eosin (for eosinophils). Mast cell and eosinophil numbers in a jejunum were evaluated in 10 sections from five mice. The representative microscopic images were obtained (Olympus DP 70, Tokyo, Japan) at ×400 magnification, HPF.

### Histamine assay

Serum was collected within 30 min after oral administration of casein and assayed for histamine by an ELISA kit according to the company’s instructions (Beckman Coulter, Brea, CA).

### Statistical analysis

The results were presented as means ± SEM (n ≥ 5). Statistical analysis was determined using the ANOVA followed by Duncan’s test or Dunn’s multiple comparisons test for multiple experimental groups. The symptom score was determined using the Wilcoxon Rank Sum test. Data was analyzed by *SigmaStat* software (Systat Software, Inc, Point Richmond, CA). In all comparisons, *p < 0.05 and **P < 0.01 were used to determine statistical significance.

## Additional Information

**How to cite this article**: Kim, A.-R. *et al*. Mesenteric IL-10-producing CD5^+^ regulatory B cells suppress cow's milk casein-induced allergic responses in mice. *Sci. Rep.* **6**, 19685; doi: 10.1038/srep19685 (2016).

## Supplementary Material

Supplementary Information
