# Supplementary material for: Mesenteric IL-10-producing CD5+ regulatory B cells suppress cow’s milk casein-induced allergic responses in mice
Source: Sci Rep. 2016 Jan 20;6:19685. doi: 10.1038/srep19685 (PMC4726293; doi:10.1038/srep19685)
Supplement: Supplementary Information [file srep19685-s1.pdf]

Supplementary information

Mesenteric IL-10-producing CD5$^+$ regulatory B cells suppress cow's milk casein-induced allergic responses in mice

A-Ram Kim[1†], Hyuk Soon Kim[1†], Do Kyun Kim[1], Seung Taek Nam[1], Hyun Woo Kim[1], Young Hwan Park[1], Dajeong Lee[1], Min Bum Lee[1], Jun Ho Lee[1], Bokyung Kim[1], Michael A. Beaven[2], Hyung Sik Kim[3], Young Mi Kim[4] & Wahn Soo Choi[1*]

[1]School of Medicine, Konkuk University, Chungju 380-701, Korea; [2]Laboratory of Molecular Immunology, National Heart, Lung, and Blood Institute, National Institutes of Health, Bethesda, MD20892; [3]School of Pharmacy, Sungkyunkwan University, Suwon 440-746, Korea; [4]College of Pharmacy, Duksung Women's University, Seoul 132-714, Korea. Correspondence and requests for materials should be addressed to W.S.C. (email: wahnchoi@kku.ac.kr)

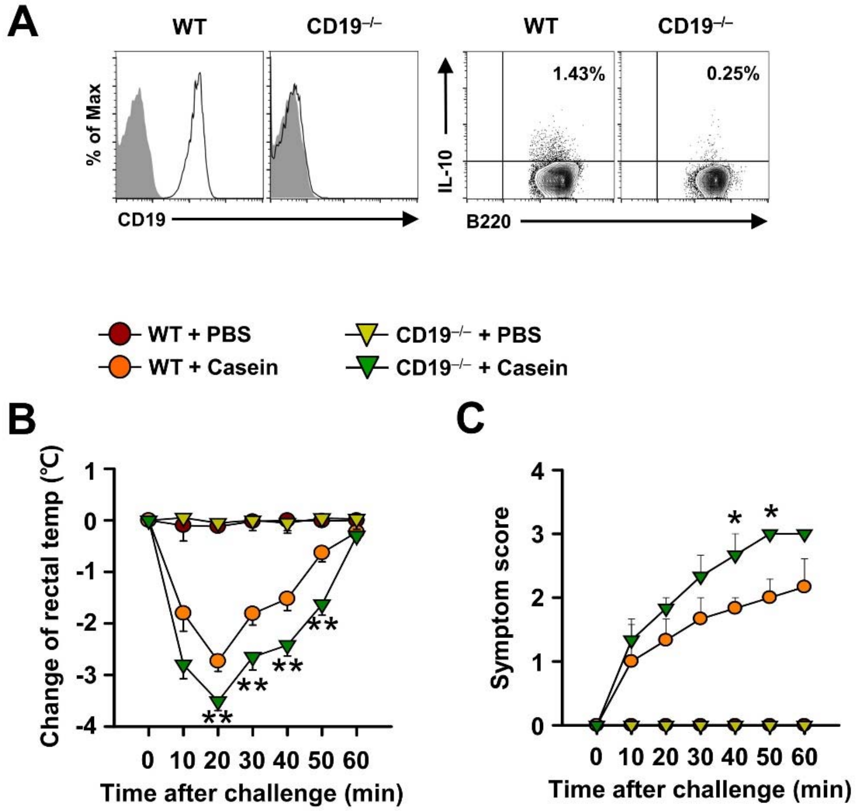

Figure S1. CD19 deficiency aggravates allergic symptoms in casein-induced allergy mice. (A) The percentage of IL-10^+ cells among $B220^+$ and $CD5^+B220^+$ MLN cells by flow cytometry. **(B)** The severity of casein-induced allergy (CIA) was estimated by changes of body temperatures and systemic symptom scores in $CD19^{-/-}$ mice. Data are mean ± SEM (n = 8). The statistical differences were presented between WT+Casein and $CD19^{-/-}$+ Casein groups (D). **, $p < 0.01$; *, $p < 0.05$; n.s., not significant.
